# Advances in Nanofluidics

**DOI:** 10.3390/mi12040427

**Published:** 2021-04-14

**Authors:** Yutaka Kazoe, Yan Xu

**Affiliations:** 1 Department of System Design Engineering, Faculty of Science and Technology, Keio University, Yokohama 223-8522, Japan; 2 Department of Chemical Engineering, Graduate School of Engineering, Osaka Prefecture University, Osaka 599-8570, Japan; 3 Japan Science and Technology Agency (JST), PRESTO, Saitama 332-0012, Japan; 4 NanoSquare Research Institute, Research Center for the 21st Century, Organization for Research Promotion, Osaka Prefecture University, Osaka 599-8570, Japan

Recently, a new frontier in fluid science and engineering at the 1 to 1000 nm scale, called nanofluidics, has developed and provided new methodologies and applications to the fields of chemistry, biology, material sciences, bioengineering, medicine, drug discovery, energy, and environmental engineering. Utilizing nanospaces, various operations at volumes in the order of atto- to femtoliters have been achieved, and novel applications are expected, such as single-molecule analysis, single-cell omics, high-efficient ion conductor/separator, high-efficient heat exchanger, and nanomaterial synthesis. Simultaneously, nanofluidics has provided research tools for the elucidation of phenomena in nanoscale confined fluids, which are important in various fields, e.g., physical chemistry, separation science, biophysics, and membrane engineering. The use of nanofluidic platforms has allowed basic experimental research under well-regulated conditions, to elucidate unique fluid properties and transport phenomena resulting from dominant surface effects owing to significantly increased surface-to-volume ratios.

The Special Issue of *Micromachines* entitled “Advances in Nanofluidics” presents a total of 10 papers, including one critical review. Two papers report fabrication technologies for nanofluidic devices, four focus on label-free detection methods, two report studies on transport phenomena in nanospaces, one presents the size sorting of exosomes utilizing a nanochannel, and one presents an antibacterial and cell-compatible surface utilizing nanowire.

A top–down fabrication-based device containing nanochannels is one of many typical device platforms in nanofluidics, which enables the integration of various chemical operations, as microfluidics has realized previously. Morikawa et al. [1] report advanced top–down fabrication processes for micro/nanofluidic devices. They achieve the precise fabrication of square-shaped nanochannels (width = depth) with defined dimensions of 10^1^ nm, which is one-order smaller than the size of square nanochannels reported previously, with a smooth connection of a nanochannel with a microchannel to construct a micro–nano size interface. These procedures are expected to allow the design of new functional devices. Shoda et al. [2] present a process of the low-temperature bonding of glass substrates with surface activation by oxygen plasma, which is available by utilizing typical facilities in clean rooms. Since the bonding of substrates is necessary to construct devices for sealing micro- and nanochannels, this work will contribute to the integration of various functional materials which do not tolerate the extremely high temperatures needed for thermal fusion, into micro/nanofluidic devices.

To achieve analytical applications utilizing micro/nanofluidic devices, a key issue is the label-free detection of ultrasmall analytes in small spaces. Le et al. [3] review recent progress in label-free detection methods for nanofluidics, which are categorized into optical detection methods (diffraction, scattering, plasmonic and photonic structure, and photothermal effect) and electrical detection methods (conductivity, electrokinetic phenomena, and electrochemical reaction). Le et al. [4] present a nanofluidic device compatible with infrared (IR) spectroscopy, which is a typical label-free detection method. The nanofluidic device is made of fused-silica and calcium fluoride, which is an ideal material for IR spectroscopy due to its transparency of IR lights. In addition, metal–insulator–metal perfect absorber metamaterials are integrated in the nanofluidic device to enhance the sensitivity by performing plasmon-enhanced IR absorption spectroscopy. Ohshiro et al. [5] present single-molecule counting by a nano-gap electrode integrated in a nanochannel. The nano-gap electrode based on a tunnel phenomenon enables the detection of individual molecules and their identification. They demonstrate the electrical detection of individual deoxyguanosine monophosphate transported by electrophoresis in the nanochannel. This work will contribute to high-throughput single molecule detection in nanofluidic devices. Liu et al. [6] report theoretical and experimental investigation on the performance of microchip-based electrochemical detection system (μEDS) utilizing a PDMS-based three-dimensional micropillar array electrode. They focus on the geometrical parameters of the micropillars and hydrodynamic parameters of microflows. The investigation not only provides some insights into the optimization of the performance of μEDS, but also would be helpful for the development of nanofluidic chip-based electrochemical detection system (nEDS) in the future.

Controlling transport phenomena in nanospaces is a fundamental issue in nanofluidics. Wang et al. [7] report a computational study on transport-induced-charge phenomena induced by the concentration imbalance between cations and anions across a thin nanopore and its influences on electroosmotic flow. They revealed that the localized temperature enhancement in the nanopore by Joule heating effects significantly affects the fluid properties and ion concentration distributions. The knowledge obtained from this work provides a useful guide for the control of fluid behavior in ultrathin nanopores. Ryuzaki et al. [8] propose and fabricate a nanopore structure to improve the sensing capabilities of nanopore-based devices. The proposed nanopore structure is an inverted pyramid (IP)-shaped structure, which enables inducing a homogeneous electric field gradient in the nanofluidic conditions within the device. The results of their study suggest that the induced homogeneous electric field gradient could improve the throughput of analytes when passing through the nanopore, and meanwhile, possess potential to reduce the analyte translocation speed, which is a critical issue in nanopore-based sensing.

Nanofluidics enables novel devices for biochemical applications by utilizing functional nanostructures. Fujiwara et al. [9] use a micro-nanofluidic device for the size-sorting of exosomes, which are a type of nanosized exocellular vesicles released from cells. The size-sorting mechanism is based on the electric double layers, which are formed within the surface of nanometers and can be tuned by adjusting the concentration of the running buffer to match the sizes of exosomes. Their experimental results suggest that the mechanism would contribute to the size separation of exosomes, which is a critical issue in exosomes-related biology and medicine. Shimada et al. [10] report the fabrication of ZnO/SiO_2_ nanowires using a hydrothermal synthesis process and a demonstration of their antibacterial performance based on a mechanical rupture mechanism. Their results indicate that the fabricated nanowires exhibit not only antibacterial activity but also compatibility with human cells. These performances are desired in the development of antibacterial surfaces, so the fabricated nanowires possess potential to be applied to antibacterial applications.

We would like to thank all authors who contribute to this Special Issue. We would also like to thank all the reviewers for their careful and timely reviews which ensured the quality of this Special Issue.
